# Investigating Stress Tolerance of Multiple *Salmonella enterica* Strains Associated With Foodborne Outbreaks

**DOI:** 10.1155/ijm/9940898

**Published:** 2026-06-04

**Authors:** A. Martin, C. Patch, V. Vanarsdall, R. Pham, G. Whitney, S. Markus, A. Lunna, A. J. Etter

**Affiliations:** ^1^ Department of Nutrition and Food Sciences, University of Vermont, Burlington, Vermont, USA, uvm.edu

**Keywords:** foodborne outbreak, *Salmonella*, stress tolerance

## Abstract

*Salmonella enterica* is a foodborne pathogen commonly found in food processing environments. Although control methods such as heat treatment and sanitizers are often used, *S. enterica* has evolved strategies for survival and persistence to overcome pathogen control. This study assessed 43 outbreak‐associated (OA) and nonoutbreak associated (NOA) *S. enterica* isolates from serovars Enteritidis, Heidelberg, Newport, Typhimurium, and monophasic Typhimurium (I 4,[5],12:i:‐) for enhanced stress tolerance. Heat shock at 56°C, minimum inhibitory concentrations (MICs) for sanitizers sodium hypochlorite (NaOCl) and peroxyacetic acid (PAA), and crystal violet microtiter assays were used to evaluate heat tolerance, sanitizer tolerance, and attachment capacity, respectively. Most isolates (*n* = 34/43) carried at least one antimicrobial resistance gene, and nearly half (*n* = 21/43) displayed genotypic and/or phenotypic resistance to ampicillin, ciprofloxacin, or ceftriaxone. Most isolates carried genes conferring resistance to gold (*n* = 43/43) and arsenic (*n* = 41/43), and tolerance to mercury, copper, and silver was common among monophasic Typhimurium and Heidelberg isolates. Efflux pump *qacEdelta1* was detected among eight Heidelberg isolates. We found enhanced stress tolerance (i.e., an unusually high ability to survive and adapt to various environmental stresses) to sanitizers and enhanced attachment capacity, indicating biofilm formation. Isolates evaluated for heat tolerance survived at least 15 min at 56°C and three survived > 60 min. Overall, we found evidence of enhanced tolerance to individual stresses across both OA and NOA *S. enterica.* There were no strong patterns based upon serovar or OA/NOA status; however, we did find that specific enhanced stress tolerance profiles may have contributed to outbreak characteristics.

## 1. Introduction

Nontyphoidal *S. enterica* (NTS) is a gram‐negative foodborne pathogen commonly found in poultry, eggs, pork, beef, nuts, and produce [2]. NTS is responsible for numerous foodborne outbreaks annually in the United States and is a common contaminant in processing facilities [3]. Salmonellosis also carries a high economic burden, with the loss of over $3.3 billion annually in healthcare costs, lost productivity, and mortality in the United States [4]. NTS can exhibit high rates of antimicrobial resistance (AMR), which is cited by the Centers for Disease Control and Prevention (CDC) as a serious public health concern [1]. More specifically, fluoroquinolone resistant NTS is considered a “high concern” pathogen by the World Health Organization [5].

To limit bacterial growth and prevent foodborne outbreaks, various pathogen control methods may be used in the food processing industry, including temperature control (e.g., heat shock) and sanitizers such as sodium hypochlorite (NaOCl) or peroxyacetic acid (PAA) [6–9]. Often, multiple techniques are used in combination to prevent the proliferation of pathogenic microorganisms [10]. Heat treatment is commonly used and highly effective [11], as *S. enterica* can grow on foods held between 4°C and 60°C (40°F–140°F; i.e., the “temperature danger zone”) [12]. In poultry processing, scalding is commonly used, either by steam‐spraying or immersion scalds, with temperature and duration varying [13]. Current U.S. Department of Agriculture Food Safety and Inspection Service (USDA‐FSIS) guidelines encompass a variety of recommendations for scalding, which vary by scald type, hard or soft, and product type, including pork, turkey, and chicken. Temperature controls are also employed in peanut and tree nut processing to reduce *S. enterica*, which include dry roasting, oil roasting, and blanching [14–16].

*S. enterica* has developed various mechanisms for survival and persistence in the food processing industry [11, 17], including sanitizer and heat tolerance, and biofilm formation [18]. Exposure to one stressor can lead to cross‐tolerance to other stressors [18], further enhancing *S. enterica*′s ability to survive and persist. For instance, heat tolerance in *S. enterica* is influenced by factors such as pre‐exposure to stress and starvation, growth phase during heat shock, and expression of heat shock proteins [19–22]. Additionally, thermal resistance mechanisms can play a role in modulating virulence [19]. Repeated exposure to antimicrobials, such as sanitizers, can lead to elevated minimum inhibitory concentration (MIC), or the level of sanitizer required to inhibit the growth of or kill the bacteria; however, development of true resistance is rare [23, 24]. *S. enterica* may also form biofilms, which are difficult to eliminate and provide protection against sanitizers, desiccation, antibiotics, and some host defenses [25–28]. These biofilms often form on untreated or mechanically sanded steel surfaces, even when dry and lacking a steady nutrient source [29]. Biofilms are an especially critical target, as an estimated 80% of all U.S. bacterial infections are linked to foodborne pathogens residing in biofilms [28]. Because these persistence mechanisms, or stress tolerances, may allow certain *S. enterica* strains to survive processing steps that typically kill pathogens, it is critical that they are thoroughly understood. This can allow for continual development of processing techniques, outbreak response, and consumer risk modeling to ultimately reduce the burden of foodborne illness [11].

Previous research by Etter et al. highlighted that six *Salmonella* Heidelberg isolates from a 2013–2014 chicken poultry outbreak displayed enhanced heat tolerance and attachment capacity under stressful conditions, which may have contributed to outbreak scope and severity [20]. That research laid the groundwork for this study, which is aimed at further investigating enhanced stress tolerances, variation by serovar, and overall mechanisms that may contribute to outbreak characteristics across multiple *S. enterica* serovars and outbreaks. [11] The objectives of this research were to characterize (i) AMR and stress‐associated genes, (ii) sanitizer tolerance, (iii) heat tolerance, and (iv) attachment capacity of *S. enterica* isolates of various serovars to understand intrinsic characteristics contributing to differences between serovars and isolates associated with historically relevant outbreaks, as well as nonoutbreak associated isolates.

## 2. Methods

### 2.1. Acquisition of Culture Collection

Forty‐three strains were included in this study (Table 1); 25 were obtained from the USDA‐ARS Culture Collection (NRRL), six from the Food and Drug Administration (FDA), three from the New York State Food Laboratory, eight from the Cornell Food Safety Lab, and one strain was ordered from the American Type Culture Collection (ATCC). Ethical approval via IACUC or IRB was not necessary for this study as it did not involve animal or human participants. Laboratory biosafety protocols were approved by the University of Vermont IBC REG201900012.

**Table 1:** Characteristics of strains used in this study.

FML strain	Obtained from^a^	Isolate identifiers	Serovar^b^	Biosample accession	CgMLST type	Outbreak	Specific source
FML‐M2‐0001	USDA‐ARS	B‐65037, B‐59140, FSIS‐FY14‐1	Heidelberg	SAMN02709124	14616	Chicken, 2013–14	Chicken tenderloin
FML‐M2‐0002	USDA‐ARS	B‐65038, B‐59141, FSIS‐FY14‐2	Heidelberg	SAMN02709125	14619	Chicken, 2013–14	Chicken thighs
FML‐M2‐0003	USDA‐ARS	B‐65039, B‐59142, FSIS‐FY14‐3	Heidelberg	SAMN0270912	14444	Chicken, 2013–14	Rotisserie chicken
FML‐M2‐0004	USDA‐ARS	B‐65040, FSIS‐FY14‐4	Heidelberg	SAMN02709127	14626	Chicken, 2013–14	Chicken breast
FML‐M2‐0005	USDA‐ARS	B‐65042, B‐59143, FSIS‐FY14‐6	Heidelberg	SAMN02709129	14621	Chicken, 2013–14	Chicken drumsticks
FML‐M2‐0006	USDA‐ARS	B‐65043, B‐59144, FSIS‐FY14‐7	Heidelberg	SAMN02709130	14624	Chicken, 2013–14	Chicken tenderloin
FML‐M2‐0007	USDA‐ARS	B‐65044, FSIS‐FY14‐8	Heidelberg	SAMN02709131	14625	Chicken, 2013–14	Chicken leg quarters
FML‐M2‐0008	USDA‐ARS	B‐65046, B‐59146, FSIS‐FY14‐10	Heidelberg	SAMN02709133	14623	Chicken, 2013–14	Raw intact chicken
FML‐M2‐0009	USDA‐ARS	B‐65047, FSIS‐FY14‐11	Heidelberg	SAMN02709134	14620	Chicken, 2013–14	Chicken drumsticks
FML‐M2‐0010	USDA‐ARS	B‐65048, FSIS‐FY14‐12	Heidelberg	SAMN02709135	14617	Chicken, 2013–14	Raw intact chicken
FML‐M2‐0011	USDA‐ARS	B‐65411, FSIS1503788	m. Typh.	SAMN04088947	10418	Roast pork, 2015	Roaster swine
FML‐M2‐0012	USDA‐ARS	B‐65412, FSIS1503789	m. Typh.	SAMN04125694	10418	Roast pork, 2015	Market swine
FML‐M2‐0013	USDA‐ARS	B‐65413, FSIS1503790	m. Typh.	SAMN04125695	8618	Roast pork, 2015	Market swine
FML‐M2‐0014	USDA‐ARS	B‐65414, FSIS1503791	m. Typh.	SAMN04125696	10421	Roast pork, 2015	Roaster swine
FML‐M2‐0015	USDA‐ARS	B‐65415, FSIS1503792	m. Typh.	SAMN04125697	8618	Roast pork, 2015	Market swine
FML‐M2‐0016	USDA‐ARS	B‐65416, FSIS1503800	m. Typh.	SAMN04088949	12051	Roast pork, 2015	Roaster swine
FML‐M2‐0017	USDA‐ARS	B‐65417, FSIS1503803	m. Typh.	SAMN04088950	12050	Roast pork, 2015	Market swine
FML‐M2‐0018	USDA‐ARS	B‐65418, FSIS1503831	m. Typh.	SAMN04088343	8558	Roast pork, 2015	Roaster swine
FML‐M2‐0019	USDA‐ARS	B‐65419, FSIS1503896	m. Typh.	SAMN04054238	8618	Roast pork, 2015	Roaster swine
FML‐M2‐0020	USDA‐ARS	B‐65420, FSIS1503955	m. Typh.	SAMN04160886	8618	Roast pork, 2015	Market swine
FML‐M2‐0021	USDA‐ARS	B‐65421, FSIS1503956	m. Typh.	SAMN04160887	8618	Roast pork, 2015	Market swine
FML‐M2‐0022	USDA‐ARS	B‐65435, FSIS1606261	m. Typh.	SAMN04856237	917	Roast pork, 2015	Pork sausage
FML‐M2‐0023	USDA‐ARS	B‐65436, FSIS1606262	m. Typh.	SAMN04856238	929	Roast pork, 2015	Ground pork
FML‐M2‐0024	USDA‐ARS	B‐65437, FSIS1606267	m. Typh.	SAMN04855045	911	NOA, 2016	Sausage
FML‐M2‐0025	USDA‐ARS	B‐65439, FSIS1606643	m. Typh.	SAMN05001758	39962	NOA, 2016	Pork feet
FML‐M2‐0026	FDA	N42462, 12CT11GT04‐S	Heidelberg	SAMN05201972	46955	NOA, 2012	Ground turkey
FML‐M2‐0027	FDA	N15798, 07NM11GT07‐S	Heidelberg	SAMN03842331	11444	NOA, 2007	Ground turkey
FML‐M2‐0028	FDA	N37936, 11NY10CB10‐S	Enteritidis	SAMN05201730	47354	NOA, 2011	Chicken breast
FML‐M2‐0029	FDA	N30688, 11NY04CB01‐S	Typh. v. 5‐	SAMN05201583	47184	NOA, 2011	Chicken breast
FML‐M2‐0030	NYSAGM	11B09799A‐1, CFSAN062518	Heidelberg	SAMN06672001	8100	Chicken liver, 2011	Fresh chopped chicken liver
FML‐M2‐0031	NYSAGM	11B09801A‐1, CFSAN062519	Heidelberg	SAMN06672000	8100	Chicken liver, 2011	Broiled chicken liver
FML‐M2‐0032	NYSAGM	11B09887A‐1, CFSAN062520	Heidelberg	SAMN06671998	8100	Chicken liver, 2011	Broiled chicken liver
FML‐M2‐0033	ATCC	ATCC BAA‐1045	Enteritidis	SAMN07682529	170461	Almonds, 2003–04	Raw almonds
FML‐M2‐0036	Cornell	FSL S10‐1621; CFSAN068898	Enteritidis	SAMN08048784	35137	NOA, 2011	Produce farm soil
FML‐M2‐0037	Cornell	FSL S10‐1623	Enteritidis	SAMN01902470	35137	NOA, 2011	Produce farm soil
FML‐M2‐0038	Cornell	FSL S10‐1644	Enteritidis	SAMN01902476	35137	NOA, 2011	Produce farm swab
FML‐M2‐0040	Cornell	FSL R9‐5251; MDD314R	Newport	SAMN07411290	170519	NOA	Tomatoes
FML‐M2‐0041	Cornell	FSL R9‐5252; MDD314	Newport	SAMN07411291	170750	NOA	Tomatoes
FML‐M2‐0043	Cornell	FSL S10‐1020	Newport	SAMN01902454	13323	NOA	Farm, drag swab
FML‐M2‐0044	Cornell	FSL S10‐1060	Newport	SAMN01902455	35117	NOA	Farm, soil
FML‐M2‐0046	Cornell	FSL S10‐1630	Newport	SAMN01902472	35133	NOA	Water
FML‐M2‐0047	FDA	SAL 9672	Typh.	SAMN02678704	17662	Peanut butter, 2008–09	Peanut paste
FML‐M2‐0048	FDA	SAL 10566	Typh.	SAMN02846344	4207	Peanut butter, 2008–09	Peanut butter cheese cracker

^a^Obtained from: USDA‐ARS = USDA‐ARS Culture Collection, NYSAGM = New York State Department of Agriculture and Marketing, Cornell = Cornell Food Safety Laboratory.

^b^Serovar: m. Typh. =monophasic Salmonella Typhimurium is I, 4,[5],12:i‐., Typh. v. 5‐ = Typhimurium variant 5‐, Typh. = Typhimurium.

Upon acquisition, cultures were transferred into sterile trypticase soy broth (TSB; BD, Franklin Lakes, New Jersey) and incubated (37°C, 200 rpm, 24 h), then streaked out for isolation onto trypticase soy agar (TSA; BD, Franklin Lakes, New Jersey) and incubated (37°C, 24 h). Isolates were stored in sterile cryovials in 25% glycerol at –80°C as a working stock for future use.

### 2.2. Sanitizer Tolerance

MICs for sodium hypochlorite (NaOCl) and PAA sanitizer tolerances were determined as follows: using a procedure adapted from methods previously described [20, 30]. Due to limited access to PAA sanitizer, assays were performed on only a subset of isolates.

• *Preparation of cultures:* Isolates were recovered from working stock solutions; approximately 15–25 isolated colonies per strain were selected via sterile swab and suspended in 3 mL of TSB. Optical density at 600 nm (OD_600_) was read and adjusted to 0.600–0.800. Adjusted culture was diluted 1:100 into 1 mL of either 2X TSB or 1/10X TSB to achieve a final concentration of 1X (nutrient abundance) or 1/20X (nutrient depletion) upon sanitizer addition. An NaOCl solution (4.275% NaOCl; Clorox, Oakland, California) was prepared to achieve a concentration of 400 mg/L (400 ppm, 0.04%), then serially diluted into phosphate buffered saline (PBS) to 6.25–200 mg/L (6.25–200 ppm, 0.000625–0.02%) upon addition of bacterial culture. A PAA solution (Inspexx 250, Saint Paul, Minnesota) was prepared to a concentration of 400 mg/L (400 ppm, 0.04%) and serially diluted 1:1 into PBS to 25–200 mg/L (25–200 ppm, 0.0025%–0.02%) upon addition of bacterial cultures.
• *Plate inoculation and incubation:* Polystyrene microtiter plates were prepared at 1/20X and 1X conditions per strain in triplicate. Plates were read immediately following inoculations to determine a baseline OD_600_ and read again at 24 h to measure growth at room temperature (22°C), where the MIC was the concentration of sanitizer at which no growth occurred in the wells.

### 2.3. Heat Shock

Growth curves and heat shock assays were performed to determine heat tolerance using procedures adapted from methods previously described by Etter et al. [20]. A subset of 11 isolates were tested, representing four serovars from five outbreaks. Heat tolerance differences among strains from respective serovars and outbreaks was assessed when applicable. Isolates were recovered from cryopreserved samples (−80°C), inoculated on TSA, and incubated (37°C, 24 h). A single colony was inoculated into 10 mL TSB and incubated (37°C, 200 rpm, 16 h) before serial dilution in duplicate by a factor of 10^−5^ and incubation (37°C, 200 rpm, 8 h). For growth curves, 1 mL aliquots were collected every 1 h for 8 h and serially diluted into PBS. Dilutions were spread‐plated onto TSA in duplicate and incubated (37°C, 36 h). For heat shock, 1 mL aliquots in 2 mL microcentrifuge tubes were transferred to preheated 56°C water baths (hard scald temperature [20, 31]) for heat shock, staggering incubation by 3 min per strain. At 0, 3, 6, 9, 15, 30, 45, and 60 min, 1 mL aliquots were removed and serially diluted into PBS. Dilutions were immediately pour‐plated with 10 mL liquid TSA in duplicate. At 15, 30, 45, and 60 min, nondiluted cultures were pour‐plated in 100 *μ*L aliquots in duplicate and 250 *μ*L aliquots in quadruplicate to capture low titer cultures, and plates were incubated (37°C, 36 h). Colonies were counted using a countable range of 30–300 colonies/plate, all counts from 250 *μ*L plates were used, and the limit of detection was 1 CFU/mL. Values were averaged across biological replicates and log transformed (log10 (CFU/mL)) prior to statistical analyses. *D-*values were determined for the initial phase of the survival curve using the first‐order kinetic model: log(*N*/*N*_0_ ) = −*t*/(*D*).

Log‐linear timepoints of 0–9 min were used, to ensure comparability across isolates showing early die off. Observed survival at later timepoints indicates persistence and heat tolerance. SE (standard error) values were used to estimate uncertainty.

### 2.4. Biofilm Assays

Attachment to polystyrene plates was evaluated using a crystal violet staining procedure adapted from methods previously described [20, 30]. Polystyrene 96‐well plates were prepared under four conditions in triplicate per strain: 1/20X TSB, 4°C (nutrient depletion, refrigeration); 1/20X TSB, RT (22°C) (nutrient depletion, room temperature); 1X TSB, 4°C (nutrient abundance, refrigeration); and 1X TSB, RT (nutrient abundance, room temperature). Absorbance at 600 nm was recorded to determine crystal violet (CV) retention as an approximation of cell density (biomass) with an Epoch microplate spectrophotometer at 24, 72, and 120 h (Agilent Technologies, Santa Clara, California). Growth was not confirmed via enumeration. All CV biofilm assays were repeated in their entirety in triplicate.

### 2.5. Genomic Characteristics

AMR genes, select stress tolerance genes, and heavy metal resistance genes for each isolate were extracted from the NCBI Isolates Browser platform, which detected these genes using AMRFinderPlus (v3.8.4) [32]. A single nucleotide polymorphism (SNP)‐based phylogenetic tree was created to assess isolate relationships using CSIPhylogeny (v1.4) [33].

### 2.6. Statistical Methods

All significant differences for the crystal violet attachment assays, the MIC assays, and the heat shock assays were assessed using analysis of variance (ANOVA), followed by post hoc pairwise comparisons using Tukey′s honestly significant difference (HSD) test in R v4.2.1 [34]. For both tests, *α* was equal to 0.05, with *p*‐values being reported for ANOVA and adjusted *p*‐values (*p_adj_*) being reported for Tukey HSD.

## 3. Results and Discussion

### 3.1. Genotypic Profiles

A SNP‐based phylogenetic tree of all isolates, aligned against reference *Salmonella* Heidelberg SL476, is available in Figure S1. All *Salmonella* Heidelberg genomes, both outbreak and nonoutbreak associated isolates, were within 130 SNPs of each other. Isolates from the kosher broiled chicken liver [35, 36] outbreak were zero SNPs different from each other, whereas isolates from the 2013–2014 poultry outbreak, which involved six strains [37], were 1–122 SNPs different. Monophasic *Salmonella* Typhimurium isolates were 1–102 SNPs different, including the NOA isolate. The two *Salmonella* Typhimurium isolates from the 2008–2009 peanut butter outbreak [38] were 73 SNPs different, despite sharing the same two‐enzyme PFGE patterns (JPXX01.0459/JPXA26.0462). The *Salmonella* Enteritidis almond outbreak isolate [39] was the most different from its paired NOA isolates; although the NOA isolates were 44–45 SNPs different from each other, they were 2440–2449 SNPs different from the OA isolate. This may have been due to source; the NOA isolates were from produce and soil.

### 3.2. Sanitizer Tolerance

#### 3.2.1. Sodium hypochlorite (NaOCl)

**Figure 1:**
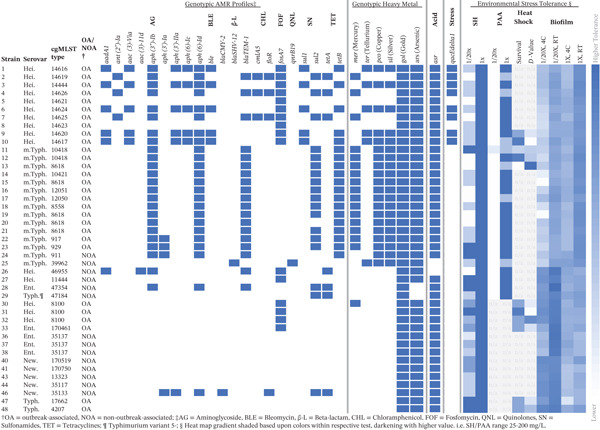
Heatmap of isolate antimicrobial resistance profiles, relevant genotypic characteristics, and results of environmental stress tolerance experiments. Heatmap values for environmental stress tolerance are based upon MIC for sanitizers, absorbance values for biofilm formation (OD_600_), and heat shock: both the timepoint to which isolates survived 56°C heat shock (30, 45, 60 or > 60 min), and the *D*‐value (min) of the initial log‐linear death curve. Shades darken as values increase within respective tests, that is, the darkest shade within SH and PAA columns indicates > 200 mg/L (ppm), whereas the lightest shades indicates 25 mg/L (ppm).

Acquired tolerance to sanitizers used in food processing environments can contribute to survival and persistence of *S. enterica* [40], increasing the risk of potential outbreaks. Exposure to sublethal doses can lead to bacterial repair mechanisms and allow for subsequent survival in extreme conditions [40]. Sanitizer tolerances are shown in Figure 1 with detailed values in Table S2. NaOCl MIC averaged > 200 mg/L in 1X TSB and 113 mg/L in 1/20X TSB, indicating that nutrient concentration influences MIC values (*p* < 0.05). MIC varied by serovar (*p* < 0.05), with serovar Typhimurium having the highest MIC (> 200 mg/L), followed by Enteritidis (125 mg/L), Heidelberg (109 mg/L), Newport (111 mg/L), and monophasic Typhimurium and Typhimurium var. 5‐ (100 mg/L). Typhimurium var. 5‐ isolates had a higher MIC than monophasic Typhimurium averaged across both conditions (*p*_adj_ < 0.05). Isolates from the roast pork (FML‐M2‐0011 through FML‐M2‐0023), chicken (FML‐M2‐0001 through FML‐M2‐0010), kosher broiled chicken liver (FML‐M2‐0030 through FML‐M2‐0032), raw almond (FML‐M2‐0033), and peanut butter (FML‐M2‐0047 through FML‐M2‐0048) outbreaks had mean NaOCl MIC values at 1/20X TSB of 92, 95, 158, 150, and > 200 mg/L, respectively, though differences were not significant (*p*_adj_ > 0.05). NOA isolates had higher mean MIC values in 1/20X (125 mg/L) when compared with OA isolates (106 mg/L), though the difference between was not significant (*p* < 0.05).

Previous studies have also reported high NaOCl tolerance among *S. enterica* isolates [41–44]. Xiao et al. found > 256 mg/L MIC when using 11.92% NaOCl for most poultry supply chain‐derived isolates (*n* = 161/172) [41], whereas Obe et al. observed MIC values of 500–1,000 mg/L using 12.5% NaOCl [42]. Humayoun et al. reported a mean MIC of 3,152 mg/L [43] with a lower active chlorine concentration (5.25%–6.15% NaOCl). Sublethal NaOCl concentrations (50 mg/L) were marginally more effective against Heidelberg than Typhimurium and Enteritidis isolates, though nonsignificant [45], consistent with this study.

Higher NaOCl tolerance has been linked to AMR and the *qacEdelta1* efflux pump [41]; however, Heidelberg isolates in this study carrying both (*n* = 8; Figure 1) had a lower mean MIC than other isolates. The high and moderate MIC values of nut‐associated isolates (> 200 mg/L in 1X, Typhimurium FML‐M2‐0047 and FML‐M2‐0048, OA, peanut butter; 150 mg/L in 1X, Enteritidis FML‐M2‐0033, OA, almond) may be due to their low‐moisture food matrices, which have been associated with cross‐tolerance to NaOCl [46]. The concerningly high NaOCl tolerance observed in this study and others may contribute to *S. enterica* persistence in the food processing environments where this sanitizer is used.

#### 3.2.2. PAA

PAA MIC values for isolates FML‐M2‐0001 through FML‐M2‐0029 averaged 176 mg/L in 1X TSB and 26 mg/L in 1/20X TSB; isolates FML‐M2‐0030 through FML‐M2‐0048 were not tested. In 1/20X TSB, all isolates had MIC values of 25 mg/L, except for monophasic Typhimurium FML‐M2‐0011 (50 mg/L; OA, roast pork). Nutrient concentration influenced MIC values (*p* < 0.05), with nearly all isolates exhibiting higher MIC values in 1X TSB than 1/20X (*p*_adj_ < 0.05). MICs varied little by serovar or outbreak, remaining between 25–27 mg/L in 1/20X and 170–200 mg/L in 1X TSB. Monophasic Typhimurium had the lowest MIC in 1X (173 mg/L), whereas Enteritidis and Typhimurium var. 5‐ had the highest (> 200 mg/L). However, serovar was not a significant determinant of MIC (*p* > 0.05). By outbreak status, the lowest MIC in 1X TSB was among Heidelberg chicken outbreak isolates (170 mg/L), whereas NOA isolates had the highest (183 mg/L), though differences were not significant (*p*_adj_ > 0.05). Similarly to NaOCl, outbreak status (NOA or OA) did not influence PAA tolerance (*p* < 0.05). Overall, PAA tolerance was lower than NaOCl, though nutrient concentration appeared more influential in PAA tolerance.

PAA tolerance findings aligned with previous studies. Etter et al. reported an average MIC of 73.6 mg/L among MDR (multidrug resistant) *Salmonella* Heidelberg isolates from the 2013–2014 chicken outbreak testing concentrations of 25–250 mg/L [20]. Mourao et al. found an MIC of 60–70 mg/L in *S. enterica* from chicken meat using 5–90 mg/L PAA [47]. In contrast, Jolivet‐Gougeon et al. reported an MIC of 7 mg/L PAA for *Salmonella* Typhimurium LT2 [48]. Humayoun et al. found a much higher average MIC of 880 mg/L in 88 MDR *S. enterica* isolates using PAA concentrations of 80–15,104 *μ*g ml^−1^ [43], but found no association between MDR and increased PAA tolerance. Micciche et al. reported MICs of 500 mg/L for household PAA (62.5–4000 mg/L), and 1000 mg/L for industrial grade PAA (62.5–4000 mg/L) in *Salmonella* Typhimurium derived from animals [49].

*Salmonella* Typhimurium can withstand potentially lethal acid shock (pH < 4.0) following adaptation to milder acidic conditions [50], which may partially explain the high MIC values among Typhimurium isolates. Acid tolerance can also be partially enhanced by pre‐exposure to other stressors [51], which may be more prevalent in food processing environments [52]. This study did not evaluate PAA tolerance among nonfood processing derived isolates (i.e., soil, water, and farm swabs: FML‐M2‐0036 through FML‐M2‐0038 and FML‐M2‐0043, FML‐M2‐0044, and FML‐M2‐0046), limiting evaluation of food processing stress effects on PAA tolerance. It is important to note that MIC values observed in all previous studies were below the 2000 mg/L PAA limit set by the USDA‐FSIS for poultry processing water (as a dip, spray, rinse, and chill) [53], though advisable concentrations may vary by product and specific manufacturer′s specifications for use. These findings emphasize the importance of using sanitizers at full working concentrations to avoid tolerance and avoid inducing AMR [54].

### 3.3. Heat Shock

**Figure 2:**
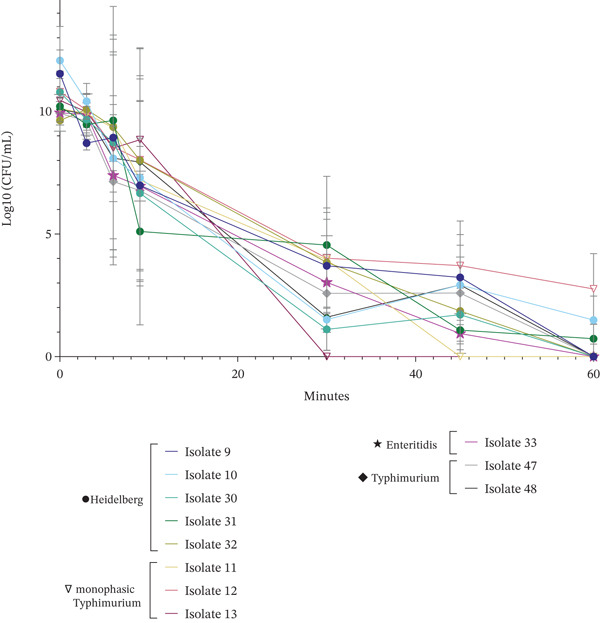
Heat tolerance of isolates (*n* = 11) in stationary phase undergoing heat shock at 56°C. Time for isolates to reach 56°C from 37°C incubation (i.e., come‐up time) was 2 min, 21 s.

Development of heat tolerance can allow for bacterial survival of *S. enterica* through food processing and potentially incomplete cooking, as well as provide cross‐protection to other stresses [21]. We previously identified unusual heat tolerance in an outbreak‐associated *S. enterica* serovar Heidelberg, including a strain associated with illness from ready‐to‐eat rotisserie chicken [20, 37]. Consequently, we investigated whether this might be a common strategy among the outbreak‐associated isolates in this study. We assessed the heat tolerance of 11 OA isolates (FML‐M2‐0009 through FML‐M2 0013, FML‐M2‐0030 through FML‐M2‐0033, FML‐M2‐0047, and FML‐M2‐0048) from four serovars at 56°C (Figure 2, Table 2) after conducting growth curves (data not shown) to confirm comparability of growth profiles. All isolates survived at least 15 min of heat shock, 10 survived past 30 min, nine past 45 min, and three past 60 min. Isolates showed a biphasic survival curve, with an initial log‐linear reduction, followed by a plateau or flattening of the curve, indicating a heat‐tolerant subpopulation which was previously reported for *Salmonella* Enteritidis PT4 by Humpheson et al. [22]. The most heat tolerant isolates (Heidelberg FML‐M2‐0010, OA, chicken and monophasic Typhimurium FML‐M2‐0012, OA, roast pork) had log‐10 transformed values of 31 CFU/mL and 575 CFU/mL, respectively, after 60 min. Serovar (*p* < 0.05) and sampling time (*p* < 0.05) influenced heat tolerance, but individual isolate number did not (*p* > 0.05). Bacterial counts (CFU/mL) did not significantly decrease after 3 min at 56°C (*p*_adj_ > 0.05) but decreased after 6 min (*p*_adj_ < 0.05). Enteritidis isolate (FML M2‐0033, OA, raw almonds) was more heat tolerant than monophasic Typhimurium (FML‐M2‐0011 through FML‐M2‐0013, OA, roast pork; *p*_adj_ < 0.05) and Typhimurium isolates (FML M2‐0047 and FML M2‐0048, OA, peanut butter; *p*_adj_ < 0.05). *D-*Values were calculated for the initial log‐linear section of the biphasic survival curve (Table 2). These *D-*values ranged from 1.42 to 4.25 min, with FML‐M2‐0012 having the highest *D-*value and FML‐M2‐0010 having the lowest. By serovar, monophasic Typhimurium had the highest average *D-*value at 3.04 min, followed by Typhimurium, Heidelberg, and Enteritidis which had *D-*values of 2.33, 2.06, and 2.02 min, respectively. *D-*values, indicative of initial die‐off, were not mutually exclusive with observed survival of the heat‐tolerant subpopulation: isolates with survival at 60 min (Heidelberg FML‐M2‐0010, OA, chicken; Heidelberg FML‐M2‐0031, OA, broiled chicken liver; and monophasic Typhimurium FML‐M2‐0012, OA, roast pork) had initial *D-*values of 1.42, 1.48, and 2.47 min, respectively.

**Table 2:** Subset of isolates heat tolerance data was collected from. Initial *D*‐values are indicative of rapid thermal inactivation of the log‐linear portion of the biphasic curve (sampling times 0–9 min).

Isolate	Serovar	Food product	*D* − *v* *a* *l* *u* *e* ± *S* *E* (min)	*R* ^2^
FML‐M2‐0009	Heidelberg	Chicken	1.92 ± 0.83	0.73
FML‐M2‐0010	Heidelberg	Chicken	1.42 ± 0.39	0.87
FML‐M2‐0011	monophasic Typhimurium	Roast pork	2.39 ± 0.52	0.91
FML‐M2‐0012	monophasic Typhimurium	Roast pork	2.47 ± 0.50	0.92
FML‐M2‐0013	monophasic Typhimurium	Roast pork	4.25 ± 2.40	0.61
FML‐M2‐0030	Heidelberg	Chicken liver	1.79 ± 0.27	0.96
FML‐M2‐0031	Heidelberg	Chicken liver	1.48 ± 0.57	0.77
FML‐M2‐0032	Heidelberg	Chicken liver	3.70 ± 1.11	0.85
FML‐M2‐0033	Enteritidis	Almonds	2.02 ± 0.46	0.91
FML‐M2‐0047	Typhimurium	Peanut butter	1.97 ± 0.46	0.90
FML‐M2‐0048	Typhimurium	Peanut butter	2.69 ± 0.69	0.88

Survival of poultry‐associated strains at 56°C is concerning, where scalds in poultry processing typically last < 2 min at 51°C–54°C for soft scalds and 59°C–64°C for hard scalds [55]. Three isolates from the 2013–2014 chicken outbreak (FML‐M2‐0002/R1‐002, FML‐M2‐0005/R1‐0004, FML‐M2‐0008/R1‐0007) previously displayed enhanced heat tolerance [20], whereas only FML‐M2‐0010 did in the present study. However, nearly all strains survived beyond typical scald durations, indicated by *D*‐values exceeding 2 min among all but two isolates (Table 2). Discrepancies between *D-*values and observed survival times are indicative of heat‐tolerant subpopulations. Inadequate cooking, as noted in the kosher broiled liver outbreak (FML‐M2‐0030 through FML‐M2‐0032 in Figure 2) [36], and possibly the roast pork (FML‐M2‐0011 through FML‐M2‐0013 [56]) and chicken outbreaks (FML‐M2‐0009 and FML‐M2‐0010 [37, 57]), may have contributed to survival. Dawoud et al. reported that sublethal heat exposure can enhance bacterial thermal resistance, suggesting that prior heat exposure during food processing or cooking could have contributed to the survival observed in these outbreaks [19]. Results from in vitro heat shock assays in this study support this hypothesis.

Burns et al. found comparable heat tolerance in monophasic Typhimurium isolates from pig feed production, with one highly heat tolerant strain with the ASSuT AMR profile (resistances to ampicillin [A], streptomycin [S], sulfisoxazole [Su], and tetracycline [T]); isolates with the same AMR profile were implicated in the roast pork outbreak (Table S1) [56, 58]. However, only two of three isolates exhibiting heat tolerance at 60 min (FML‐M2‐0010 and FML‐M2‐0012) were MDR, and prior research found no consistent AMR‐heat tolerance association among poultry outbreak Heidelberg isolates [20].

Although the heat tolerance of peanut butter and raw almond isolates was high, this temperature is substantially lower than that used in nut processing (> 95°C) [14–16]. Previous studies have demonstrated *S. enterica* survival while dry roasting peanuts at temperatures up to 120°C [59], and thermal resistance in peanut butter is well documented [60]. Ma et al. found that *Salmonella* Tennessee outbreak strains survived for 50 min at 90°C, with a 1‐log CFU/g reduction, and were only undetectable after 120 min [61]. Similarly, Shachar and Yaron observed that peanut butter derived isolates of *Salmonella* Agona, Enteritidis and Typhimurium only had a 3.2‐log reduction after 50 min at 90°C, with even lower efficacy at 80°C and 70°C [62]. Furthermore, *Salmonella* in dry products, such as nuts, are more heat resistant [63]. We found contradictory thermal resistance among isolates from dry products; the raw almond isolate FML‐M2‐0033 was more tolerant compared with peanut butter isolates FML‐M2‐0047 and FML‐M2‐0048 (*p* < 0.05). Regulations regarding almond processing changed following the almond outbreaks to require a minimum of 4‐log CFU reduction of *Salmonella* from heat treatments [64], necessitated following large influxes of illness. Previous studies have observed variation in heat tolerance by serovar and strain, similar to findings in this study. Two studies reported that *Salmonella* Enteritidis isolates were generally more resistant than Typhimurium [6, 63], whereas another study did not find differences by serovar substantial [6]. Hosts with higher internal body temperatures (e.g., chicken: 42°C) may activate thermal stress resistance mechanisms [19], which may explain enhanced heat tolerance among poultry‐derived Heidelberg. Enhanced survival has also been observed in human illness‐associated Enteritidis PT4 strains [65]. Lastly, we found potential cross‐tolerances between heat stress and other environmental stresses. Induction of heat tolerance is known to provide subsequent cross‐resistance to other stresses [21, 50], and we found that heat tolerant Heidelberg isolate FML‐M2‐0031 (OA, kosher broiled chicken liver) survived past 60 min of scald at 56°C, attached well in 1/20X TSB at 22°C, and also had an NaOCl MIC ≥ 200 mg/L. Heidelberg isolate FML‐M2‐0010 (OA, chicken) survived past 60 min of heat shock and attached strongly in 1X TSB at 22°C, but did not display additional phenotypic stress tolerances. Monophasic Typhimurium isolate FML‐M2‐0012 (OA, roast pork) had the highest CFU/mL after 60 min of heat shock and displayed enhanced attachment capacity but had relatively low NaOCl tolerance (50 mg/L in 1/20X, 200 mg/L in 1X). It is important to note that following heat exposure, test tubes in this experiment were not immersed in cold water, which could have prolonged thermal stress.

Overall, isolates′ enhanced heat tolerance presents a concern for food processing facilities. According to the USDA‐FSIS *Salmonella* Framework for Raw Poultry Products, a product is considered adulterated if it contains 10 CFU/mL or more of a serovar of public health concern [66]. After 60 min of a 56°C heat shock significantly longer than used in processing for most products, we found two isolates were still above this level of contamination (Heidelberg FML‐M2‐0010, OA, chicken and monophasic Typhimurium FML‐M2‐0012, OA, roast pork), which is extremely concerning, though in vitro tests′ comparability to raw poultry products may be limited.

### 3.4. Biofilm Assays

**Figure 3:**
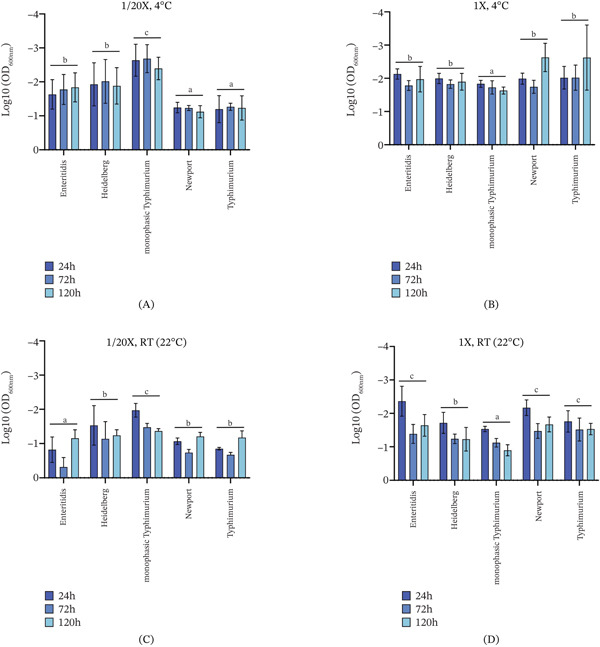
Attachment capacity of isolates, grouped by serovar, on polystyrene plates measured by crystal violet at 24, 72, and 120 h in (A) 1/20X 4°C; (B) 1X 4°C; (C) 1/20X RT; and (D) 1X RT conditions, where a Log10(OD_600_) value closer to zero (shorter bar) indicates higher biomass and thus better attachment capacity. Within each condition, bars, grouped by serovar, indicated with the same letter are not significantly different from each other, whereas bars indicated by a different letter show significant differences between serovars (Tukey′s HSD, p_adj_ < 0.05).

As the majority (80%) of bacterial infections in the United States are linked to foodborne pathogens residing in biofilms [28] and biofilms have been involved in several foodborne outbreaks [67–70], understanding attachment and biofilm formation capacity is crucial to identifying potential attributes to outbreaks. We evaluated all isolates at 24 h, 72 h, and 120 h for biomass (OD_600_) as an indication of attachment capacity and biofilm formation under four conditions: 1/20X 4°C (nutrient depletion, refrigeration); 1/20X RT (22°C) (nutrient depletion, room temperature); 1X 4°C (nutrient abundance, refrigeration); and 1X RT (22°C) (nutrient depletion, room temperature) (Figure 3).

In 1/20X 4*°*C, biomass varied by serovar (*p* < 0.05), with Newport and Typhimurium having the greatest biomass, Enteritidis and Heidelberg being moderate, and monophasic Typhimurium with the least (*p*_adj_ < 0.05) (Figure 3). Isolates Heidelberg FML‐M2‐0031 (OA, kosher broiled chicken liver) and Typhimurium FML‐M2‐0048 (OA, peanut butter) had the highest biomass (*p*_adj_ < 0.05) (Figure 3). Biomass was unaffected by sampling time (24, 72, or 120 h) (*p* < 0.05). In 1X TSB, 4°C, biomass varied by serovar, sampling time, isolate, and serovar‐time interactions (*p* < 0.05). Biomass peaked at 72 h for all serovars except monophasic Typhimurium, which peaked at 120 h, outperforming all other serovars (*p*_adj_ < 0.05) (Figure 3). Monophasic Typhimurium isolates had the highest biomass compared with all other serovars over all sampling times (*p*_adj_ < 0.05) (Figure 3), and Typhimurium FML‐M2‐0012 (OA, roast pork) had a particularly high biomass (*p*_adj_ < 0.05). NOA isolates had greater biomass at 72 h than OA isolates, but OA isolates had greater biomass at 24 and 120 h, though not significant.

At 1/20X RT, biomass varied by serovar, outbreak, time, isolate number, and serovar‐time interactions (*p* < 0.05). Enteritidis had the highest biomass (*p*_adj_ < 0.05), whereas monophasic Typhimurium had the lowest (*p*_adj_ < 0.05). Biomass was highest at 72 h (*p*_adj_ < 0.05). At 72 h, Enteritidis had particularly high biomass (*p*_adj_ < 0.05), whereas monophasic Typhimurium had particularly low biomass, but exceeded all other serovars at 120 h (Figure 3). NOA isolates tended to form stronger biofilms compared with OA isolates, though not significant. In 1X TSB RT, biomass varied by serovar, outbreak, sampling time, isolate number, and serovar‐time interactions (*p* < 0.05). Biomass was highest at 120 h compared with 72 h (*p*_adj_ < 0.05). Monophasic Typhimurium had the highest biomass across all serovars (*p*_adj_ < 0.05). OA isolates had higher biomass at all sampling times compared with NOA isolates (*p* < 0.05). Heidelberg isolates had higher biomass than Enteritidis, Newport, and Typhimurium (*p*_adj_ < 0.05). The three kosher broiled chicken liver outbreak Heidelberg isolates (FML‐M2‐0030 through FML‐M2‐0032) had lower biomass compared with chicken outbreak Heidelberg isolates (FML‐M2‐0001 through FML‐M2‐0010). Specifically, FML‐M2‐0003 outperformed eight other isolates (*p*_adj_ < 0.05) (Figure 3).

Overall, biomass was highest in 1/20X RT, followed by 1X RT. By serovar, Typhimurium isolates had the highest mean biomass in all conditions, followed by Newport, Enteritidis, Heidelberg, and monophasic Typhimurium. Monophasic Typhimurium isolates had the lowest biomass in 1/20X TSB, but the highest in 1X TSB. Enteritidis isolates had particularly high biomass values in 1X RT. Serovars Newport and Typhimurium also had particularly high biomass values in the high stress conditions of 1/20X 4°C. At room temperature, biomass at 72 and 120 h exceeded 24 h, suggesting that initial attachment is less temperature‐dependent and maturation of biofilms is enhanced at RT compared with 4°C. OA isolates had greater biomass in 1X, RT at all sampling times (*p*_adj_ < 0.05) and marginally greater biomass in 1X, 4°C at 24 and 120 h (*p* > 0.05). NOA isolates had higher biomass in 1/20X, 4°C at all sampling times (*p*_adj_ < 0.05), marginally higher biomass in 1/20X RT at all sampling times, and marginally higher biomass in 1X 4°C at 72 h.

Consistent with findings in this study, Stepanović et al. found that *Salmonella* biofilm formation in 1/20X TSB was more effective than 1X TSB across 122 strains [71]*. Salmonella*′s ability to form biofilms in response to starvation stress [72] is concerning, as 1/20X TSB conditions mimic the food processing industry [71]. Additionally, Stepanović et al. found that isolate source does not impact biofilm forming ability [71], which contradicts findings in this study where differences in biomass in 1X TSB were influenced by outbreak (*p*_adj_ < 0.05), though serovar distribution may be confounding. Agarwal et al. tested 151 strains across 69 serovars and found that over half formed moderate or strong biofilms [73]. Enteritidis outperformed Typhimurium in 1X 4°C, consistent with results from this study, and the highest biomass was observed at 48 h and 72 h, respectively [73]. Interestingly, Burns et al. noted biofilm formation as a potential future direction of research in their study evaluating heat tolerance among monophasic Typhimurium isolates from pig feed [58]. This study found that monophasic Typhimurium had both the highest *D-*values by serovar, as well as the highest attachment capacity in both 1X conditions. These enhanced tolerance characteristics could have contributed to persistence observed in the 2015 roast pork outbreak, as biofilm formation is crucial for environmental survival [74]. Further investigation into swine associated monophasic Typhimurium biofilm capacity is warranted. Lastly, Wang et al. linked sanitizer tolerance to biofilm forming ability [75]. In this study, Typhimurium, the strongest biofilm formers across all conditions, consistently exhibited the highest NaOCl tolerance, whereas the one Typhimurium isolate tested (FML‐M2‐0029) had the highest PAA tolerance, along with Enteritidis isolates. Key findings determined that attachment and biofilm forming capacity varied widely by serovar and conditions, with seemingly favorable conditions by strain; and overall enhanced biofilm formation in lower nutrient availability conditions, which mimics the food processing environment, warrants concern. Eliminating biofilms remains a key challenge in the food industry, and understanding *Salmonella* strain specificity is an opportunity to reduce bacterial persistence and outbreak potential.

### 3.5. AMR Genes

AMR is a concern for *S. enterica* outbreaks as resistance to medically relevant antibiotics (defined as ampicillin, ciprofloxacin, azithromycin, ceftriaxone, and amoxicillin) can complicate the treatment of severe clinical cases and AMR genes often travels on plasmids which can carry virulence, AMR, and sanitizer efflux genes [1, 76–79]. We were interested in whether AMR might be connected to stress tolerance in outbreak‐associated *S. enterica.*

Most isolates (*n* = 34/43) carried at least one gene conferring AMR (Figure 1). Nearly half (*n* = 21/43) harbored genes associated with resistance to at least one medically important antibiotic. Three Heidelberg isolates from the 2013–2014 chicken outbreak and all monophasic Typhimurium isolates from the 2015 roast pork outbreak carried *blaTEM-1,* conferring ampicillin resistance. Additionally, *blaTEM-1* was detected in one NOA isolate each from serovars Heidelberg, monophasic Typhimurium, and Enteritidis. Newport isolate FML‐M2‐0046 (NOA, water) contained *blaCMY-2*, linked to third‐generation cephalosporin resistance [80, 81]. NOA monophasic Typhimurium isolate FML‐M2‐0025 (NOA, pork feet) contained *blaSHV-12* (linked to ampicillin and cephalosporin resistance) [82] and *qnrB19* (intermediate quinolone resistance, defined as 0.12–0.5 mg/L [83]); it was the only isolate to carry either gene. Phenotypic resistance data (Table S1, available for a limited number of isolates) showed that monophasic Typhimurium isolate FML‐M2‐0025 (NOA, pork feet) also exhibited intermediate ciprofloxacin resistance and Enteritidis isolate FML‐M2‐0028 (NOA, chicken breast) exhibited intermediate resistance to amoxicillin/clavulanic acid. As for genotypic resistance among NOA isolates, half (*n* = 7/14) contained no AMR genes, whereas AMR genes were particularly prevalent among Heidelberg isolate FML‐M2‐0026 (NOA, ground turkey), Enteritidis isolate FML‐M2‐0028 (NOA, chicken breast), and Newport isolate FML‐M2‐0046 (NOA, water).

Efflux pump *qacEdelta1*, encoding a quaternary ammonium compound (QAC) efflux pump, was detected in eight Heidelberg chicken outbreak isolates (FML‐M2‐0001 through FML‐M2‐0004, FML‐M2‐0006, FML‐M2‐0007, FML‐M2‐0009, and FML‐M2‐0010). This gene has been linked to tolerance to QACs, and it may be selected for in environments where QAC‐based sanitizers are used [84, 85]. The *asr* gene (anaerobic sulfite reduction), encoding an acid shock protein for survival in acidic conditions (such as acid‐based antimicrobials [86]), was present in all but two NOA isolates (Enteritidis FML‐M2‐0028, NOA, raw chicken breast; Typhimurium var. 5‐ FML‐M2‐0029, NOA, chicken breast).

Higher resistance levels in some OA strains have been previously reported; Etter et al. found 1–10 AMR genes among seven Heidelberg strains from the 2013–2014 chicken outbreak [20], whereas phenotypic testing of 2015 roast pork outbreak monophasic Typhimurium strains showed most were multidrug resistant [56]. Conversely, studies show lower resistance levels among food‐derived isolates that are not specifically OA. Procura et al. found few AMR genes in *S. enterica* from chicken livers, except erythromycin resistance in all isolates and streptomycin resistance in 22% [87]. Furthermore, in a study comparing prevalence of AMR in *Salmonella* from animal and nut products, the authors found most *S. enterica* from raw almonds (*n* = 73/83) were resistant to two or fewer of 15 antimicrobials, with 52 being pan susceptible [88]. Overall, OA isolates in this study carried a higher number of AMR genes when compared with NOA isolates, but AMR gene carriage did not show a clear tie to increased heat tolerance, attachment, or sanitizer tolerance.

### 3.6. Heavy Metal Tolerance Genes

Carriage of heavy metal tolerance genes has been previously linked with carriage of AMR genes in *Salmonella* isolated from both humans and livestock; these genes may be carried on plasmids or other transferable elements [89–92]. Consequently, we assessed carriage of heavy metal resistance genes in the evaluated isolates. Carriage of genes conferring gold and arsenic tolerance was nearly ubiquitous among *S. enterica* isolates (Figure 1). All contained *golST* for gold tolerance, and all but two (Enteritidis FML‐M2‐0028, NOA, chicken breast; Typhimurium var. 5‐ FML‐M2‐0029, NOA, chicken breast) carried *arsR* or *arsABCDR* for arsenic tolerance. Copper (*pcoABCDERS* or *pcoACDRS*) and silver (*silABCEFPRS*) genes were found in all monophasic Typhimurium isolates (FML‐M2‐0011 through FML‐M2‐0025) and five 2013–2014 chicken outbreak Heidelberg isolates (FML‐M2‐0001, FML‐M2‐0003, FML‐M2‐0006, FML‐M2‐0009, and FML‐M2‐0010). Tellurium tolerance (*terDWZ*) was present in these five Heidelberg isolates and monophasic Typhimurium isolate FML‐M2‐0025 (NOA, pork feet). Mercury tolerance (*merAPR*, *merABDPRT*, or *merACDEPRT*) was observed in three chicken outbreak Heidelberg isolates (FML‐M2‐0002, FML‐M2‐0004, and FML‐M2‐0007), all roast pork monophasic Typhimurium isolates, and one kosher broiled chicken liver outbreak Heidelberg isolate (FML‐M2‐0030). Mercury tolerance did not co‐occur with tellurium, copper, or silver tolerance among Heidelberg isolates, nor with tellurium tolerance among monophasic Typhimurium isolates.

Overall, monophasic Typhimurium isolates carried the greatest number of heavy metal tolerance genes, consistent with previous research [93]. Acquisition of these genes facilitates bacterial survival and has been noted to contribute to outbreak severity [93]. Furthermore, the highest frequency of both heavy metal tolerance and AMR genes was observed in Heidelberg isolates from the chicken outbreak and monophasic Typhimurium isolates from the roast pork outbreak, which was previously reported for *S. enterica* from livestock, animal foods and carcass samples in the EU, where heavy metals are often used as growth promoters [89]. Nonetheless, heavy metal tolerance gene carriage did not show a clear tie to increased heat tolerance, attachment, or sanitizer tolerance.

### 3.7. Outbreak Status

Overall, we found no strong evidence to support that isolate outbreak status alone (OA or NOA) is associated with enhanced stress tolerance profiles; rather, the combination of specific outbreak characteristics and *S. enterica* strain was determinant of phenotypic tolerances. This has been found among *E. coli* isolates [94]. However, expanding research to include more NOA from a higher variety of sources could strengthen this research area. Limitations of this study constrained the ability to generalize results and their applicability. For heat shock, *D*‐value calculations are of limited utility when applied to biphasic, nonlinear death curves with long survivor “tails,” which complicated interpretation of the heat tolerance assay results. Furthermore, due to resource constraints, only subsets of isolates were tested for PAA tolerance, heat shock, and phenotypic AMR, which limited our ability to perform robust cross tolerance analyses. Nonetheless, this study provided valuable phenotypic stress tolerance information on for a large set of isolates from multiple *Salmonella* serovars, food sources, and outbreaks.

## 4. Conclusions

We assessed 43 OA and NOA *S. enterica* isolates from various serovars for phenotypic stress tolerance to heat and sanitizers, and for attachment capacity under processing‐relevant conditions (nutrient abundance vs. depletion, and room temperature vs. refrigeration). Isolates displayed varying patterns of stress tolerance, with some showing potential cross‐tolerance to multiple stresses (heat stress, sanitizer tolerance, and biofilm). Thermal resistance indicated by survival in lethal temperatures was shown by at least one meat and poultry outbreak isolate, suggesting that heat tolerance could potentially have contributed to these outbreaks, particularly in cases of improper consumer preparation. Nut‐associated isolates also exhibited enhanced stress tolerance: in low nutrient conditions, *Salmonella* Typhimurium and *Salmonella* Enteritidis isolates showed enhanced biofilm formation and NaOCl tolerance, with the latter also exhibiting the highest heat tolerance across all time points. *S. enterica* serovar was more influential in the observed tolerances, rather than whether the isolate was NOA or OA. Overall, these isolates had phenotypic characteristics, which could have contributed to the scope and severity of their respective outbreaks, emphasizing the importance of continual improvement in food processing techniques. Further research regarding the complex and nuanced relationship between specific *S. enterica* strains, stress tolerance, and their impacts to human health is needed to accurately model risks and best support regulations in the food processing industry.

## Author Contributions

Martin A. and Patch C. contributed equally to this work.

## Funding

This study was supported by USDA National Institute of Food and Agriculture (NIFA) Agriculture and Food Research Initiative Strengthening and New Investigator Food Safety and Defense Program (2019‐06903); USDA Hatch funding mechanism via the Vermont Agricultural Experiment Station and gift funds provided to the Department of Animal and Veterinary Sciences at the University of Vermont for C. Patch′s contributions.

## Disclosure

This work has been made available as a preprint on BioRxiv [95].

## Conflicts of Interest

The authors declare no conflicts of interest.

## Supporting Information

Additional supporting information can be found online in the Supporting Information section.

## Supporting information


**  Supporting Information 1** Figure S1: Phylogenetic tree of isolates included in this study.

**  Supporting Information 2** Table S1: Phenotypic AMR profiles for isolates in this study (from NCBI/isolate metadata).

**  Supporting Information 3** Table S2: Tolerance to sodium hypochlorite (NaOCl) and peroxyacetic acid (PAA) as minimum inhibitory concentrations (MIC; ppm).

## Data Availability

The data that support the findings of this study are available from the corresponding author upon reasonable request.

## References

[1] CDC , Antibiotic Resistance Threats in the United States, 2019, https://www.cdc.gov/antimicrobial-resistance/data-research/threats/?CDC_AAref_Val=https://www.cdc.gov/drugresistance/Biggest-Threats.html.

[2] White P. L. , Naugle A. L. , Jackson C. R. , Fedorka-Cray P. J. , Rose B. E. , Pritchard K. M. , Levine P. , Saini P. K. , Schroeder C. M. , Dreyfuss M. S. , Tan R. , Holt K. G. , Harman J. , and Buchanan S. , *Salmonella* Enteritidis In Meat, Poultry, And Pasteurized Egg Products Regulated By The U.S. Food Safety And Inspection Service, 1998 Through 2003, Journal of Food Protection. (2007) 70, no. 3, 582–591, 10.4315/0362-028X-70.3.582, 17388045. 17388045

[3] WHO , Salmonella (Non-Typhoidal), 2018, https://www.who.int/en/news-room/fact-sheets/detail/salmonella-.

[4] Hoffmann S. , Batz M. B. , and Morris J. G. , Annual Cost Of Illness And Quality-Adjusted Life Year Losses In The United States Due To 14 Foodborne Pathogens, Journal of Food Protection. (2012) 75, no. 7, 1292–1302, 10.4315/0362-028X.JFP-11-417, 22980013. 22980013

[5] WHO , Salmonella (Non-Typhoidal), 2018, https://www.who.int/news-room/fact-sheets/detail/salmonella.

[6] Lianou A. and Koutsoumanis K. P. , Evaluation of the Strain Variability of *Salmonella enterica* Acid and Heat Resistance, Food Microbiology. (2013) 34, no. 2, 259–267, 10.1016/j.fm.2012.10.009, 23541192. 23541192

[7] Kim J.-M. , Zhang B.-Z. , and Park J.-M. , Comparison of Sanitization Efficacy of Sodium Hypochlorite and Peroxyacetic Acid Used as Disinfectants in Poultry Food Processing Plants, Food Control. (2023) 152, 109865, 10.1016/j.foodcont.2023.109865.

[8] Sun S. H. , Kim S. J. , Kwak S. J. , and Yoon K. S. , Efficacy of Sodium Hypochlorite and Acidified Sodium Chlorite in Preventing Browning and Microbial Growth on Fresh-Cut Produce, Preventive Nutrition and Food Science. (2012) 17, no. 3, 210–216, 10.3746/pnf.2012.17.3.210, 24471086. 24471086 PMC3866743

[9] Sithole T. R. , Ma Y. X. , Qin Z. , Wang X. D. , and Liu H. M. , Peanut Butter Food Safety Concerns—Prevalence, Mitigation and Control of *Salmonella* spp., and Aflatoxins in Peanut Butter, Foods. (2022) 11, no. 13, 10.3390/foods11131874, 35804689. PMC9265579 35804689

[10] Leistner L. , Basic Aspects of Food Preservation by Hurdle Technology, International Journal of Food Microbiology. (2000) 55, no. 1-3, 181–186, 10.1016/S0168-1605(00)00161-6, 10791741. 10791741

[11] Pye H. V. , Thilliez G. , Acton L. , Kolenda R. , al-Khanaq H. , Grove S. , and Kingsley R. A. , Strain and Serovar Variants of *Salmonella enterica* Exhibit Diverse Tolerance to Food Chain-Related Stress, Food Microbiology. (2023) 112, 104237, 10.1016/j.fm.2023.104237, 36906307. 36906307

[12] USDA-FSIS , How Temperatures Affect Food, 2020, https://www.fsis.usda.gov/food-safety/safe-food-handling-and-preparation/food-safety-basics/how-temperatures-affect-food#:%7E:text=Cold%2520Storage%2520Temperatures-,The%20%22Danger%20Zone%22%20(40,%C2%B0F%2D140%20%C2%B0F)&text=This%20range%20of%20temperatures%20is,of%20refrigeration%20over%202%20hours.

[13] USDA-FSIS , FSIS Guideline for Controlling *Salmonella* in Raw Poultry, 2021, https://www.fsis.usda.gov/guidelines/2021-0005.

[14] Almond Board of California , Guidelines for Validation of Dry Roasting Processes, 2007, https://www.almonds.com/sites/default/files/2020-05/dry-roast-validation-guidelines.pdf.

[15] Almond Board of California , Guidelines for Validation of Oil Roasting Processes, 2007, https://www.almonds.com/sites/default/files/2020-05/oil-roast-validation-guidelines.pdf.

[16] Almond Board of California , Guidelines for Validation of Blanching Processes, 2007, https://www.almonds.com/sites/default/files/content/attachments/blanching-validation-guidelines.pdf.

[17] Guillén S. , Nadal L. , Álvarez I. , Mañas P. , and Cebrián G. , Impact of the Resistance Responses to Stress Conditions Encountered in Food and Food Processing Environments on the Virulence and Growth Fitness of Non-Typhoidal *Salmonella*e, Foods. (2021) 10, no. 3, 10.3390/foods10030617, 33799446. PMC8001757 33799446

[18] Humphrey T. , *Salmonella*, Stress Responses and Food Safety, Nature Reviews Microbiology. (2004) 2, no. 6, 504–509, 10.1038/nrmicro907. 15152206

[19] Dawoud T. M. , Davis M. L. , Park S. H. , Kim S. A. , Kwon Y. M. , Jarvis N. , O’Bryan C. A. , Shi Z. , Crandall P. G. , and Ricke S. C. , The Potential Link Between Thermal Resistance and Virulence in *Salmonella*: A Review, Frontiers in Veterinary Science. (2017) 4, 10.3389/fvets.2017.00093, 28660201. PMC5469892 28660201

[20] Etter A. J. , West A. M. , Burnett J. L. , Wu S. T. , Veenhuizen D. R. , Ogas R. A. , and Oliver H. F. , *Salmonella enterica* Subsp. *enterica* Serovar Heidelberg Food Isolates Associated With A Salmonellosis Outbreak Have Enhanced Stress Tolerance Capabilities, Applied and Environmental Microbiology. (2019) 85, no. 16, e01065-19, 10.1128/AEM.01065-19, 31175193. 31175193 PMC6677849

[21] Humphrey T. J. , Richardson N. P. , Statton K. M. , and Rowbury R. J. , Effects of Temperature Shift on Acid and Heat Tolerance in *Salmonella* Enteritidis Phage Type 4, Applied and Environmental Microbiology. (1993) 59, no. 9, 3120–3122, 10.1128/aem.59.9.3120-3122.1993, 8215381. 8215381 PMC182415

[22] Humpheson L. , Adams M. R. , Anderson W. A. , and Cole M. B. , Biphasic Thermal Inactivation Kinetics in *Salmonella* Enteritidis PT4, Applied and Environmental Microbiology. (1998) 64, no. 2, 459–464, 10.1128/AEM.64.2.459-464.1998, 9464380. 9464380 PMC106066

[23] Krewing M. , Mönch E. , Bolten A. , and Niesalla H. , Resistance or Tolerance? Highlighting the Need for Precise Terminology in the Field of Disinfection, Journal of Hospital Infection. (2024) 150, 51–60, 10.1016/j.jhin.2024.05.006. 38782058

[24] Cerf O. , Carpentier B. , and Sanders P. , Tests for Determining in-Use Concentrations of Antibiotics and Disinfectants Are Based on Entirely Different Concepts: "Resistance" Has Different Meanings, International Journal of Food Microbiology. (2010) 136, no. 3, 247–254, 10.1016/j.ijfoodmicro.2009.10.002, 19853944. 19853944

[25] Stepanovic S. , Cirkovic I. , Ranin L. , and Svabic-Vlahovic M. , Biofilm Formation by *Salmonella* spp. and *Listeria monocytogenes* on Plastic Surface, Letters in Applied Microbiology. (2004) 38, no. 5, 428–432, 10.1111/j.1472-765X.2004.01513.x, 15059216. 15059216

[26] Jahid I. K. and Ha S.-D. , The Paradox of Mixed-Species Biofilms in the Context of Food Safety, Comprehensive Reviews in Food Science and Food Safety. (2014) 2014, no. 13, 990–1011, 10.1111/1541-4337.12087.

[27] Corcoran M. , Morris D. , de Lappe N. , O′Connor J. , Lalor P. , Dockery P. , and Cormican M. , Commonly Used Disinfectants Fail to Eradicate *Salmonella enterica* Biofilms From Food Contact Surface Materials, Applied and Environmental Microbiology. (2014) 80, no. 4, 1507–1514, 10.1128/AEM.03109-13, 24362427. 24362427 PMC3911063

[28] Steenackers H. , Hermans K. , and De Keersmaecker S. C. J. , *Salmonella* biofilms: An Overview on Occurrence, Structure Regulation and Eradication, Food Research International. (2012) 2012, no. 45, 502–531, 10.1016/j.foodres.2011.01.038.

[29] Schlisselberg D. B. and Yaron S. , The Effects Of Stainless Steel Finish On *Salmonella* Typhimurium Attachment, Biofilm Formation And Sensitivity To Chlorine, Food Microbiology. (2013) 35, no. 1, 65–72, 10.1016/j.fm.2013.02.005, 23628616. 23628616

[30] Wang J. , Ray A. J. , Hammons S. R. , and Oliver H. F. , Persistent and Transient *Listeria monocytogenes* Strains From Retail Deli Environments Vary in Their Ability to Adhere and Form Biofilms and Rarely Have inlA Premature Stop Codons, Foodborne Pathogens and Disease. (2015) 12, no. 2, 151–158, 10.1089/fpd.2014.1837, 25569840. 25569840

[31] McKee S. R. , Townsend J. C. , and Bilgili S. F. , Use of a Scald Additive to Reduce Levels of *Salmonella* Typhimurium During Poultry Processing, Poultry Science. (2008) 87, no. 8, 1672–1677, 10.3382/ps.2008-00061, 18648065. 18648065

[32] Feldgarden M. , Brover V. , Gonzalez-Escalona N. , Frye J. G. , Haendiges J. , Haft D. H. , Hoffmann M. , Pettengill J. B. , Prasad A. B. , Tillman G. E. , Tyson G. H. , and Klimke W. , AMRFinderPlus and the Reference Gene Catalog Facilitate Examination of the Genomic Links Among Antimicrobial Resistance, Stress Response, and Virulence, Scientific Reports. (2021) 11, no. 1, 12728, 10.1038/s41598-021-91456-0, 34135355. 34135355 PMC8208984

[33] Kaas R. S. , Leekitcharoenphon P. , Aarestrup F. M. , and Lund O. , Solving the Problem of Comparing Whole Bacterial Genomes Across Different Sequencing Platforms, PLoS One. (2014) 9, no. 8, e104984, 10.1371/journal.pone.0104984, 25110940. 25110940 PMC4128722

[34] R Core Team , R: A Language and Environment for Statistical Computing, 2022, R Foundation for Statistical Computing.

[35] CDC , 2011 *Salmonella* Outbreak Linked to “Kosher Broiled Chicken Livers” From Schreiber Processing Corporation, 2012, http://cdc.gov/www_cdc_gov/salmonella/2011/chicken-liver-1-11-2012.html.

[36] Lanier W. A. , Hale K. R. , Geissler A. L. , and Dewey-Mattia D. , Chicken Liver-Associated Outbreaks of Campylobacteriosis and Salmonellosis, United States, 2000-2016: identifying Opportunities for Prevention, Foodborne Pathogens and Disease. (2018) 15, no. 11, 726–733, 10.1089/fpd.2018.2489, 30192164. 30192164 PMC6247982

[37] Gieraltowski L. , Higa J. , Peralta V. , Green A. , Schwensohn C. , Rosen H. , Libby T. , Kissler B. , Marsden-Haug N. , Booth H. , Kimura A. , Grass J. , Bicknese A. , Tolar B. , Defibaugh-Chávez S. , Williams I. , Wise M. , and Salmonella Heidelberg Investigation Team , National Outbreak of Multidrug Resistant *Salmonella* Heidelberg Infections Linked to a Single Poultry Company, PLoS One. (2016) 11, no. 9, e0162369, 10.1371/journal.pone.0162369, 27631492. 27631492 PMC5025200

[38] Cavallaro E. , Date K. , Medus C. , Meyer S. , Miller B. , Kim C. , Nowicki S. , Cosgrove S. , Sweat D. , Phan Q. , Flint J. , Daly E. R. , Adams J. , Hyytia-Trees E. , Gerner-Smidt P. , Hoekstra R. M. , Schwensohn C. , Langer A. , Sodha S. V. , Rogers M. C. , Angulo F. J. , Tauxe R. V. , Williams I. T. , Behravesh C. B. , and Salmonella Typhimurium Outbreak Investigation Team , *Salmonella* Typhimurium Infections Associated With Peanut Products, New England Journal of Medicine. (2011) 365, no. 7, 601–610, 10.1056/NEJMoa1011208, 21848461. 21848461

[39] CDC , Outbreak of *Salmonella* Serotype Enteritidis Infections Associated With Raw Almonds -- United States and Canda, 2003-2004, Morbidity And Mortality Weekly Report. (2004) 53, no. 22, 484–487, https://www.cdc.gov/mmwr/preview/mmwrhtml/mm5322a8.htm. 15190247

[40] Obe T. , Kiess A. S. , and Nannapaneni R. , Antimicrobial tolerance in *Salmonella*: Contributions to Survival and Persistence in Processing Environments, Animals. (2024) 14, no. 4, 10.3390/ani14040578, 38396546. PMC10886206 38396546

[41] Xiao X. , Bai L. , Wang S. , Liu L. , Qu X. , Zhang J. , Xiao Y. , Tang B. , Li Y. , Yang H. , and Wang W. , Chlorine Tolerance and Cross-Resistance to Antibiotics in Poultry-Associated *Salmonella* Isolates in China, Frontiers in Microbiology. (2022) 12, 10.3389/fmicb.2021.833743. PMC8854976 35185838

[42] Obe T. , Nannapaneni R. , Schilling W. , Zhang L. , and Kiess A. , Antimicrobial Tolerance, Biofilm Formation, and Molecular Characterization of *Salmonella* Isolates From Poultry Processing Equipment, Journal of Applied Poultry Research. (2021) 30, no. 4, 100195, 10.1016/j.japr.2021.100195.

[43] Humayoun S. B. , Hiott L. M. , Gupta S. K. , Barrett J. B. , Woodley T. A. , Johnston J. J. , Jackson C. R. , and Frye J. G. , An Assay for Determining the Susceptibility of *Salmonella* Isolates to Commercial and Household Biocides, PLoS One. (2018) 13, no. 12, e0209072, 10.1371/journal.pone.0209072, 30571686. 30571686 PMC6301668

[44] Aljuwayd M. , Malli I. A. , Ricke S. C. , and Kwon Y. M. , Reactive Oxygen Species Mediate the Bactericidal Activity of Chlorine Against *Salmonella* , Current Microbiology. (2024) 81, no. 11, 10.1007/s00284-024-03880-w, 39278982. 39278982

[45] Aljuwayd M. , Olson E. G. , Abbasi A. Z. , Rothrock M. J. , Ricke S. C. , and Kwon Y. M. , Potential Involvement of Reactive Oxygen Species in the Bactericidal Activity of Eugenol Against *Salmonella* Typhimurium, Pathogens. (2024) 13, no. 10, 10.3390/pathogens13100899, 39452770. PMC11510353 39452770

[46] Gruzdev N. , Pinto R. , and Sela S. , Effect of Desiccation on Tolerance of *Salmonella enterica* to Multiple Stresses, Applied and Environmental Microbiology. (2011) 77, no. 5, 1667–1673, 10.1128/AEM.02156-10, 21216905. 21216905 PMC3067256

[47] Mourão J. , Rebelo A. , Ribeiro S. , Peixe L. , Novais C. , and Antunes P. , Atypical non-H2S-Producing Monophasic *Salmonella* Typhimurium ST3478 Strains From Chicken Meat At Processing Stage Are Adapted to Diverse Stresses, Pathogens. (2020) 9, no. 9, 10.3390/pathogens9090701, 32859122. PMC7557518 32859122

[48] Jolivet-Gougeon A. , Sauvager F. , Bonnaure-Mallet M. , Colwell R. R. , and Cormier M. , Virulence of viable But Nonculturable *S.* Typhimurium LT2 After Peracetic Acid Treatment, International Journal of Food Microbiology. (2006) 112, no. 2, 147–152, 10.1016/j.ijfoodmicro.2006.06.019, 16876276. 16876276

[49] Micciche A. C. , Feye K. M. , Rubinelli P. M. , Lee J. A. , Knueven C. J. , and Ricke S. C. , Comparison of Acid Sanitizers on *Salmonella* Typhimurium Inoculated Commercial Poultry Processing Reuse Water, Frontiers in Sustainable Food Systems. (2019) 2, 10.3389/fsufs.2018.00090.

[50] Wesche A. M. , Gurtler J. B. , Marks B. P. , and Ryser E. T. , Stress, Sublethal Injury, Resuscitation, and Virulence of Bacterial Foodborne Pathogens, Journal of Food Protectio. (2009) 72, no. 5, 1121–1138, 10.4315/0362-028X-72.5.1121, 19517746. 19517746

[51] Dubois-Brissonnet F. , Naïtali M. , Mafu A. A. , and Briandet R. , Induction of Fatty Acid Composition Modifications and Tolerance to Biocides in *Salmonella enterica* Serovar Typhimurium by Plant-Derived Terpenes, Applied and Environmental Microbiology. (2011) 77, no. 3, 906–910, 10.1128/AEM.01480-10, 21131520. 21131520 PMC3028700

[52] Galán-Relaño Á. , Valero Díaz A. , Huerta Lorenzo B. , Gómez-Gascón L. , Mena Rodríguez M. Á. , Carrasco Jiménez E. , Pérez Rodríguez F. , and Astorga Márquez R. J. , *Salmonella* and salmonellosis: An Update on Public Health Implications and Control Strategies, Animals. (2023) 13, no. 23, 10.3390/ani13233666, 38067017. PMC10705591 38067017

[53] USDA-FSIS , List of Approved On-Line Reprocessing (OLR) Antimicrobial Systems for Poultry, 2022, https://www.fsis.usda.gov/policy/fsis-directives/7120.1.

[54] Donaghy J. A. , Jagadeesan B. , Goodburn K. , Grunwald L. , Jensen O. N. , Jespers A. , Kanagachandran K. , Lafforgue H. , Seefelder W. , and Quentin M. C. , Relationship of Sanitizers, Disinfectants, and Cleaning Agents With Antimicrobial Resistance, Journal of Food Protection. (2019) 82, no. 5, 889–902, 10.4315/0362-028X.JFP-18-373, 31021666. 31021666

[55] US Dept of Agriculture, Food Safety & Inspection Service , Guideline for Controlling *Salmonella* in Raw Poultry, 2021, https://www.fsis.usda.gov/guidelines/2021-0005.

[56] Kawakami V. , Bottichio L. , Lloyd J. , Carleton H. , Leeper M. , Olson G. , Li Z. , Kissler B. , Angelo K. M. , Whitlock L. , Sinatra J. , Defibaugh-Chavez S. , Bicknese A. , Kay M. , Wise M. E. , Basler C. , and Duchin J. , Multidrug-Resistant *Salmonella* I 4,[5],12:i:- and *Salmonella* Infantis Infections Linked to Whole Roasted Pigs From a Single Slaughter and Processing Facility, Journal of Food Protection. (2019) 82, no. 9, 1615–1624, 10.4315/0362-028X.JFP-19-048, 31441688. 31441688 PMC6957080

[57] Green A. , Defibaugh-Chavez S. , Douris A. , Vetter D. , Atkinson R. , Kissler B. , Khroustalev A. , Robertson K. , Sharma Y. , Becker K. , Dessai U. , Antoine N. , Allen L. , Holt K. , Gieraltowski L. , Wise M. , Schwensohn C. , and Food Safety and Inspection Service (FSIS) Salmonella Heidelberg Investigation Team , Intensified Sampling in Response to a *Salmonella* Heidelberg Outbreak Associated With Multiple Establishments Within a Single Poultry Corporation, Foodborne Pathogens and Disease. (2018) 15, no. 3, 153–160, 10.1089/fpd.2017.2340, 29638165. 29638165 PMC5865244

[58] Burns A. M. , Duffy G. , Walsh D. , Tiwari B. K. , Grant J. , Lawlor P. G. , and Gardiner G. E. , Survival Characteristics of Monophasic *Salmonella* Typhimurium 4,[5],12:i:- Strains Derived From Pig Feed Ingredients and Compound Feed, Food Control. (2016) 64, 105–114, 10.1016/j.foodcont.2015.12.001.

[59] Pereira A. A. M. , Prestes F. S. , Silva A. C. M. , and Nascimento M. S. , Evaluation of the Thermal Resistance of *Salmonella* Typhimurium ATCC 14028 After Long-Term Blanched Peanut Kernel Storage, LWT - Food Science and Technology. (2020) 117, 108701, 10.1016/j.lwt.2019.108701.

[60] Pelaez M. A. B. , Anapi G. R. , Bautista D. V. , Dallo M. D. P. , Libunao J. C. M. , and Gabriel A. A. , Thermal Inactivation of *Salmonella enterica* in Philippine Flowing-Type Peanut Butter, LWT - Food Science and Technology. (2020) 129, 109507, 10.1016/j.lwt.2020.109507.

[61] Ma L. , Zhang G. , Gerner-Smidt P. , Mantripragada V. , Ezeoke I. , and Doyle M. P. , Thermal Inactivation of *Salmonella* in Peanut Butter, Journal of Food Protection. (2009) 72, no. 8, 1596–1601, 10.4315/0362-028X-72.8.1596, 19722389. 19722389

[62] Shachar D. and Yaron S. , Heat tolerance of *Salmonella enterica* Serovars Agona, Enteritidis, and Typhimurium in Peanut Butter, Journal of Food Protection. (2006) 69, no. 11, 2687–2691, 10.4315/0362-028X-69.11.2687, 17133812. 17133812

[63] Doyle M. E. and Mazzotta A. S. , Review of Studies on the Thermal Resistance of *Salmonella*e, Journal of Food Protection. (2000) 63, no. 6, 779–795, 10.4315/0362-028X-63.6.779, 10852574. 10852574

[64] Lambertini E. , Danyluk M. D. , Schaffner D. W. , Winter C. K. , and Harris L. J. , Risk of Salmonellosis From Consumption of Almonds in the North American Market, Food Research International. (2012) 45, no. 2, 1166–1174, 10.1016/j.foodres.2011.05.039.

[65] Humphrey T. J. , Slater E. , McAlpine K. , Rowbury R. J. , and Gilbert R. J. , *Salmonella* Enteritidis Phage Type 4 Isolates More Tolerant of Heat, Acid, or Hydrogen Peroxide Also Survive Longer on Surfaces, Applied and Environmental Microbiology. (1995) 61, no. 8, 3161–3164, 10.1128/aem.61.8.3161-3164.1995, 7487046. 7487046 PMC167590

[66] USDA-FSIS , Proposed Regulatory Framework to Reduce *Salmonella* Illnesses Attributable to Poultry, 2022, https://www.fsis.usda.gov/inspection/inspection-programs/inspection-poultry-products/reducing-salmonella-poultry/proposed.

[67] Ward S. , Bedale W. , and Glass K. A. , *Listeria monocytogenes* Outbreaks Related to Commercially Produced Caramel Apples: Developments in Sanitation, Product Formulation, and Packaging: A Review, Journal of Food Protection. (2022) 85, no. 9, 1287–1299, 10.4315/JFP-22-069, 35666586. 35666586

[68] Russo E. T. , Biggerstaff G. , Hoekstra R. M. , Meyer S. , Patel N. , Miller B. , Quick R. , and Salmonella Agona Outbreak Investigation Team , A Recurrent, Multistate Outbreak of *Salmonella* Serotype Agona Infections Associated With Dry, Unsweetened Cereal Consumption, United States, 2008, Journal of Food Protection. (2013) 76, no. 2, 227–230, 10.4315/0362-028X.JFP-12-209, 23433369. 23433369

[69] Annous B. A. , Solomon E. B. , Cooke P. H. , and Burke A. , Biofilm Formation by *Salmonella spp*. on Cantaloupe Melons∗∗, Journal of Food Safety. (2005) 25, no. 4, 276–287, 10.1111/j.1745-4565.2005.00024.x.

[70] Brouard C. , Espié E. , Weill F. X. , Kérouanton A. , Brisabois A. , Forgue A. M. , Vaillant V. , and de Valk H. , Two Consecutive Large Outbreaks of *Salmonella enterica* Serotype Agona Infections in Infants Linked to the Consumption of Powdered Infant Formula, Pediatric Infectious Disease. (2007) 26, no. 2, 148–152, 10.1097/01.inf.0000253219.06258.23, 17259878. 17259878

[71] Stepanović S. , Cirković I. , Ranin L. , and Svabić-Vlahović M. , Biofilm Formation by *Salmonella* spp. and Listeria monocytogenes on Plastic Surface, Journal of Applied Microbiology. (2004) 38, no. 5, 428–432, 10.1111/j.1472-765X.2004.01513.x, 15059216. 15059216

[72] Solano C. , García B. , Valle J. , Berasain C. , Ghigo J. M. , Gamazo C. , and Lasa I. , Genetic Analysis of *Salmonella* Enteritidis Biofilm Formation: Critical Role of Cellulose, Molecular Microbiology. (2002) 43, no. 3, 793–808, 10.1046/j.1365-2958.2002.02802.x, 11929533. 11929533

[73] Agarwal R. K. , Singh S. , Bhilegaonkar K. N. , and Singh V. P. , Optimization of Microtitre Plate Assay for the Testing of Biofilm Formation Ability in Different *Salmonella* Serotypes, International Food Research Journal. (2011) 18, no. 4, 1493–1498.

[74] Tassinari E. et al., Microevolution of Antimicrobial Resistance and Biofilm Formation of *Salmonella* Typhimurium During Persistence on Pig Farms, Scientific Reports. (2019) 9, no. 1. 10.1038/s41598-019-45216-w PMC6586642 31222015

[75] Wang R. , Schmidt J. W. , Harhay D. M. , Bosilevac J. M. , King D. A. , and Arthur T. M. , Biofilm Formation, Antimicrobial Resistance, And Sanitizer Tolerance Of *Salmonella enterica* Strains Isolated From Beef Trim, Foodborne Pathogens and Disease. (2017) 14, no. 12, 687–695, 10.1089/fpd.2017.2319, 29035101. 29035101

[76] Acheson D. and Hohmann E. L. , Nontyphoidal Salmonellosis, Clinical Infectious Diseases. (2001) 32, no. 2, 263–269, 10.1086/318457. 11170916

[77] Sivanandy P. , Yuk L. S. , Yi C. S. , Kaur I. , Ern F. H. S. , and Manirajan P. , A Systematic Review of Recent Outbreaks and the Efficacy and Safety of Drugs Approved for the Treatment of *Salmonella* Infections, IJID Regions. (2025) 14, 100516, 10.1016/j.ijregi.2024.100516, 39866847. 39866847 PMC11758818

[78] Robertson J. , Schonfeld J. , Bessonov K. , Bastedo P. , and Nash J. H. E. , A global survey of *Salmonella* plasmids and Their Associations With Antimicrobial Resistance, Microbial Genomics. (2023) 9, no. 5, 10.1099/mgen.0.001002. PMC10272869 37200081

[79] Emond-Rheault J. G. , Hamel J. , Jeukens J. , Freschi L. , Kukavica-Ibrulj I. , Boyle B. , Tamber S. , Malo D. , Franz E. , Burnett E. , Daigle F. , Arya G. , Sanderson K. , Wiedmann M. , Slawson R. M. , Weadge J. T. , Stephan R. , Bekal S. , Gruenheid S. , Goodridge L. D. , and Levesque R. C. , The *Salmonella enterica* Plasmidome as a Reservoir of Antibiotic Resistance, Microbial Genomics. (2020) 8, no. 7, 10.3390/microorganisms8071016. PMC7409225 32650601

[80] Bortolaia V. , Hansen K. H. , Nielsen C. A. , Fritsche T. R. , and Guardabassi L. , High Diversity of Plasmids Harbouring *blaCMY-2* Among Clinical *Escherichia coli* Isolates From Humans and Companion Animals in the Upper Midwestern USA, Journal of Antimicrobial Chemotherapy. (2014) 69, no. 6, 1492–1496, 10.1093/jac/dku011, 24500191. 24500191

[81] Call D. R. , Singer R. S. , Meng D. , Broschat S. L. , Orfe L. H. , Anderson J. M. , Herndon D. R. , Kappmeyer L. S. , Daniels J. B. , and Besser T. E. , *blaCMY-2*-positive IncA/C Plasmids From *Escherichia coli* and *Salmonella enterica* are a Distinct Component of a Larger Lineage of Plasmids, Antimicrobial Agents and Chemotherapy. (2010) 54, no. 2, 590–596, 10.1128/AAC.00055-09, 19949054. 19949054 PMC2812137

[82] Nüesch-Inderbinen M. , Heyvaert L. , Cernela N. , Zurfluh K. , Biggel M. , and Stephan R. , Emergence of *bla(SHV-12)* and *qnrS1* Encoded on IncX3 Plasmids: Changing Epidemiology of Extended-Spectrum ss-Lactamases Among Enterobacterales Isolated From Broilers, Journal of Global Antimicrobial Resistance. (2023) 33, 194–200, 10.1016/j.jgar.2023.03.008, 36972753. 36972753

[83] Sfeir M. M. , Adoption of the Updated CLSI Fluoroquinolone Breakpoints for Gram-Negative Bacteria in Microbiology Laboratories, Clinical Microbiology and Infection. (2021) 27, no. 2, 308–310, 10.1016/j.cmi.2020.07.027, 32717418. 32717418

[84] Chen S. , Fu J. , Zhao K. , Yang S. , Li C. , Penttinen P. , Ao X. , Liu A. , Hu K. , Li J. , Yang Y. , Liu S. , Bai L. , and Zou L. , Class 1 Integron Carrying *qacE* *Δ*1 Gene Confers Resistance to Disinfectant and Antibiotics in *Salmonella* , International Journal of Food Microbiology. (2023) 404, 110319, 10.1016/j.ijfoodmicro.2023.110319, 37473468. 37473468

[85] Haubert L. , Zehetmeyr M. L. , Pereira Y. M. N. , Kroning I. S. , Maia D. S. V. , Sehn C. P. , Lopes G. V. , de Lima A. S. , and da Silva W. P. , Tolerance to Benzalkonium Chloride and Antimicrobial Activity of *Butia odorata* Barb. Rodr. Extract in *Salmonella* spp. Isolates From Food and Food Environments, Food Research International. (2019) 116, 652–659, 10.1016/j.foodres.2018.08.092, 30716992. 30716992

[86] Ramos-Morales F. , Acidic pH, Virulence. (2012) 3, no. 2, 103–106, 10.4161/viru.19382. 22460638

[87] Procura F. , Bueno D. J. , Bruno S. B. , and Rogé A. D. , Prevalence, Antimicrobial Resistance Profile and Comparison of Methods for the Isolation of *Salmonella* in Chicken Liver From Argentina, Food Research International. (2019) 119, 541–546, 10.1016/j.foodres.2017.08.008, 30884687. 30884687

[88] Danyluk M. D. , Jones T. M. , Abd S. J. , Schlitt-Dittrich F. , Jacobs M. , and Harris L. J. , Prevalence and Amounts of *Salmonella* Found on Raw California almonds, Journal of Food Protection. (2007) 70, no. 4, 820–827, 10.4315/0362-028X-70.4.820, 17477248. 17477248

[89] Figueiredo R. , Card R. M. , Nunez-Garcia J. , Mendonça N. , da Silva G. J. , and Anjum M. F. , Multidrug-Resistant *Salmonella enterica* Isolated From Food Animal and Foodstuff May Also be Less Susceptible to Heavy Metals, Foodborne Pathogens and Disease. (2019) 16, no. 3, 166–172, 10.1089/fpd.2017.2418, 30480469. 30480469

[90] Mustafa G. R. , Zhao K. , He X. , Chen S. , Liu S. , Mustafa A. , He L. , Yang Y. , Yu X. , Penttinen P. , Ao X. , Liu A. , Shabbir M. Z. , Xu X. , and Zou L. , Heavy Metal Resistance in *Salmonella* Typhimurium and Its Association With Disinfectant and Antibiotic Resistance, Frontiers in Microbiology. (2021) 12, 702725, 10.3389/fmicb.2021.702725, 34421860. 34421860 PMC8371916

[91] Mourão J. , Marçal S. , Ramos P. , Campos J. , Machado J. , Peixe L. , Novais C. , and Antunes P. , Tolerance to Multiple Metal Stressors in Emerging Non-Typhoidal MDR *Salmonella* serotypes: A Relevant Role for Copper in Anaerobic Conditions, Journal of Antimicrobial Chemotherapy. (2016) 71, no. 8, 2147–2157, 10.1093/jac/dkw120, 27118781. 27118781

[92] Souza S. S. R. , Turcotte M. R. , Li J. , Zhang X. , Wolfe K. L. , Gao F. , Benton C. S. , and Andam C. P. , Population Analysis of Heavy Metal and Biocide Resistance Genes in *Salmonella enterica* From Human Clinical Cases in New Hampshire United States, Frontiers in Microbiology. (2022) 13, 983083, 10.3389/fmicb.2022.983083, 36338064. 36338064 PMC9626534

[93] Bearson B. L. , Trachsel J. M. , Shippy D. C. , Sivasankaran S. K. , Kerr B. J. , Loving C. L. , Brunelle B. W. , Curry S. M. , Gabler N. K. , and Bearson S. M. D. , The Role Of *Salmonella* Genomic Island 4 In Metal Tolerance Of *Salmonella enterica* Serovar I 4,[5],12:I:- Pork Outbreak Isolate USDA15WA-1, Genes. (2020) 11, no. 11, 10.3390/genes11111291, 33142960. PMC7716197 33142960

[94] Boll E. J. , Marti R. , Hasman H. , Overballe-Petersen S. , Stegger M. , Ng K. , Knøchel S. , Krogfelt K. A. , Hummerjohann J. , and Struve C. , Turn Up the Heat-Food and Clinical *Escherichia coli* Isolates Feature Two Transferrable Loci of Heat Resistance, Frontiers in Microbiology. (2017) 8, 10.3389/fmicb.2017.00579, 28439262. PMC5383660 28439262

[95] Martin A. , Patch C. , Vanarsdall V. , Pham R. , Whitney G. , Markus S. , Lunna A. , and Etter A. J. , Stress Tolerance of Multiple *Salmonella enterica* Strains Associated with Foodborne Outbreaks, preprint, bioRxiv, 2025 10.1101/2025.04.23.649775. PMC13238261 42256053

