# Supplementary figures and images for: Investigating Stress Tolerance of Multiple Salmonella enterica Strains Associated With Foodborne Outbreaks
Source: Int J Microbiol. 2026 Jun 4;2026:9940898. doi: 10.1155/ijm/9940898 (PMC13238261; doi:10.1155/ijm/9940898)

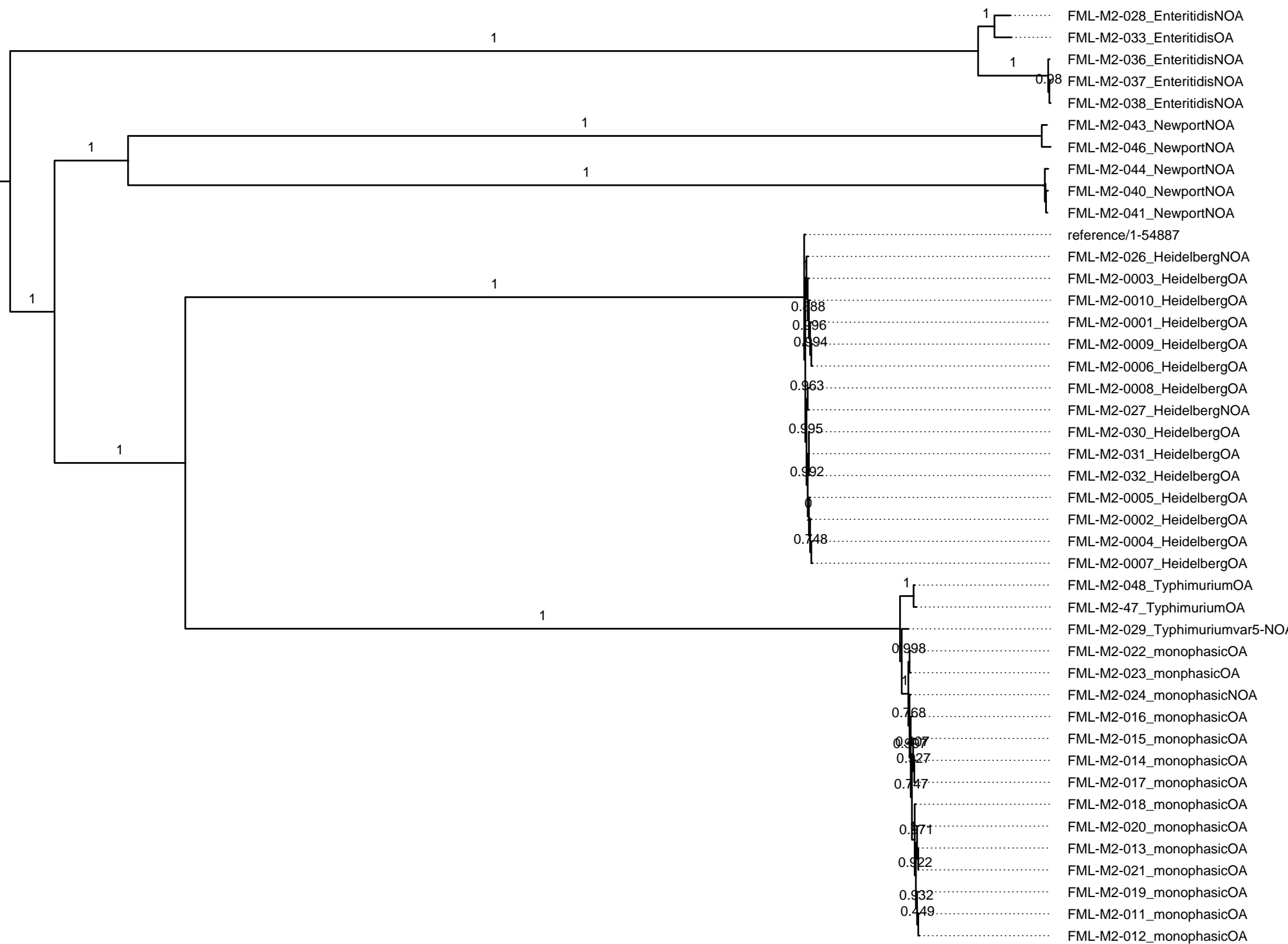

0.05

Supplement: Supplementary file 1 — Supporting Information 1 Figure S1: Phylogenetic tree of isolates included in this study. [file IJM-2026-9940898-s002.pdf]
